# Attention to language: Novel MEG paradigm for registering involuntary language processing in the brain

**DOI:** 10.1016/j.neuropsychologia.2012.07.012

**Published:** 2012-09

**Authors:** Yury Shtyrov, Marie L. Smith, Aidan J. Horner, Richard Henson, Pradeep J. Nathan, Edward T. Bullmore, Friedemann Pulvermüller

**Affiliations:** a Medical Research Council, Cognition and Brain Sciences Unit, Cambridge, UK; b Institute of Cognitive Neurology and Dementia Research, Otto-von-Guericke University, Magdeburg, Germany; c Institute of Cognitive Neuroscience, University College London, London, UK; d GlaxoSmithKline Clinical Unit, Cambridge, UK; e Brain Mapping Unit, Department of Psychiatry, University of Cambridge, Cambridge, UK; f Cognitive Brain Research Unit, Institute of Behavioural Sciences, University of Helsinki, Helsinki, Finland; g Freie Universität Berlin, Berlin, Germany; h Department of Psychological Sciences, Birkbeck College, University of London, London, UK

**Keywords:** Language, Word, Attention, ERP (ERF), MEG, EEG, Mismatch negativity (MMN), P3a

## Abstract

Previous research indicates that, under explicit instructions to listen to spoken stimuli or in speech-oriented behavioural tasks, the brain’s responses to senseless pseudowords are larger than those to meaningful words; the reverse is true in non-attended conditions. These differential responses could be used as a tool to trace linguistic processes in the brain and their interaction with attention. However, as previous studies relied on explicit instructions to attend or ignore the stimuli, a technique for automatic attention modulation (i.e., not dependent on explicit instruction) would be more advantageous, especially when cooperation with instructions may not be guaranteed (e.g., neurological patients, children etc). Here we present a novel paradigm in which the stimulus context automatically draws attention to speech. In a non-attend passive auditory oddball sequence, rare words and pseudowords were presented among frequent non-speech tones of variable frequency and length. The low percentage of spoken stimuli guarantees an involuntary attention switch to them. The speech stimuli, in turn, could be disambiguated as words or pseudowords only in their end, at the last phoneme, after the attention switch would have already occurred. Our results confirmed that this paradigm can indeed be used to induce automatic shifts of attention to spoken input. At ∼250 ms after the stimulus onset, a P3a-like neuromagnetic deflection was registered to spoken (but not tone) stimuli indicating an involuntary attention shift. Later, after the word-pseudoword divergence point, we found a larger oddball response to pseudowords than words, best explained by neural processes of lexical search facilitated through increased attention. Furthermore, we demonstrate a breakdown of this orderly pattern of neurocognitive processes as a result of sleep deprivation. The new paradigm may thus be an efficient way to assess language comprehension processes and their dynamic interaction with those of attention allocation. It does it in an automatic and task-free fashion, indicating its potential benefit for assessing uncooperative clinical populations.

## Highlights

► We suggest a new MEG paradigm to test auditory and linguistic processing in the brain. ► Initial enhanced oddball response reflects automatic neural auditory discrimination. ► P3a response only to spoken stimuli indicates involuntary attention shift to language. ► A larger pseudoword than word response reflects neural processes of lexical search. ► Different levels of auditory linguistic processing can be tested in one simple design.

## 1 Introduction

Accurate assessment of patients with cognitive and neurological impairments is often a challenge even to the most experienced clinicians. More specifically, in evaluating language function – whether for the purpose of pre-surgical mapping, to assess a child’s development, effects of brain injury or to monitor therapy progress – one has always had to rely on behavioural observation or on a combination of behavioural/verbal responses with some neuropsychological tools (e.g., Baron-Cohen, Hoekstra, Knickmeyer, & Wheelwright, 2006; Benton, 1994; Folstein, Folstein, & McHugh, 1975; Shewan & Kertesz, 1980). This raises the problem of non-cooperative subjects. A brain-damaged individual may not be able to properly respond verbally because of collateral lesion-related deficits. A young child, particularly with a speech deficit, may not be willing or able to cooperate with those assessing his or her condition. A “locked-in” person may be conscious but does not have any means to give signs of this behaviourally due to a complete absence of motor control (Laureys, Owen, & Schiff, 2004; Ragazzoni, Grippo, Tozzi, & Zaccara 2000); the range of such situations is wide. Clearly, techniques that could reveal neural correlates of language processing without relying on the individual’s overt response would be helpful in a variety of situations.

A great potential for this lies with non-invasive brain imaging techniques that have been developing rapidly in the last two decades. For instance, using functional magnetic resonance imaging (fMRI) that tracks oxygen consumption in body tissues, some studies have been able to see whether or not subjects can *mentally* (but not physically) cooperate with an instruction, giving hope for improved assessment of vegetative state and locked-in patients (for review, see Owen & Coleman, 2008). Still, it is often argued (e.g., Hagoort, 2008) that the slow temporal resolution of fMRI is not well suited to the tracking of fast neural activity related to the highly dynamic process of language processing. Indeed, while the blood-oxygenation level develops over seconds reaching maximum at ∼5–6 s (Henson, Price, Rugg, Turner, & Friston, 2002), linguistic processes operate on the temporal scale of hundreds and even tens of milliseconds (Friederici, 2002; Marslen-Wilson, 1975; Pulvermüller, Shtyrov, & Hauk, 2009). For this reason, fast neurophysiological imaging techniques like electro- or magnetoencephalography (EEG, MEG) have been used extensively in the cognitive neuroscience of language (see e.g., Friederici, 2002). These tools track mass neural activity (rather than blood metabolism) with millisecond precision, even though their spatial resolution is generally inferior to metabolic imaging (Dale & Halgren, 2001).

Still, how could one register the activity of neural networks underlying language function without having to apply linguistic tasks such as lexical decision or semantic judgements that require at least the subjects’ focussed attention and frequently involve their active participation? To address language function in cases when no active participation is possible, such tasks are clearly ruled out. To address this, a number of experiments used task-free approaches for tracing language-related neural activity. One of methodologies successfully used in such studies is the so-called passive oddball paradigm, in which the subjects are presented with linguistic contrasts between frequent repetitive stimuli and unexpected rare ones without having to perform an overt task (for reviews, see Pettigrew, Murdoch, Chenery, & Kei, 2004; Pulvermüller & Shtyrov, 2006; Shtyrov & Pulvermüller, 2007b). As no attention or stimulus-related task is required (in fact, the volunteers are distracted from the auditory input), the recorded brain activation is considered to be automatic (Näätänen, Paavilainen, Rinne, & Alho, 2007). Furthermore, because the oddball response is elicited by contrasts between the frequent and rare stimuli, this allows for full control over acoustic factors, as the same acoustic contrasts can be incorporated into linguistically different contexts (Shtyrov & Pulvermüller, 2007b).

Indeed, a series of studies using this approach established it as a sensitive tool to study the neural correlates of linguistic access including a wide range of information types: phonological, lexical, semantic and syntactic (Shtyrov & Pulvermüller, 2007b). These have shown that linguistic processing can be activated in the brain – and, importantly, this activation can be recorded in MEG/EEG – without an explicit task or an instruction to focus on the speech input. This raises the possibility of assessing neural linguistic processes non-invasively in subjects who are unable to carry out an active experimental task.

One such obvious linguistic process, utilised routinely in human communication, is so-called lexical access, i.e., access to information about individual words stored in the mental lexicon (a concept defining the store of words in a person’s mind, Aitchison, 2002). Lexical access has often been studied using non-attend auditory oddball presentation. The vast majority of such studies have uniformly shown that long-term memory traces for words can become automatically activated in the brain whenever a given word is presented in the spoken input, even if it is not specifically attended to (Pulvermüller and Shtyrov (2006), Shtyrov and Pulvermüller (2007b)). This activation manifests itself as an enhanced early (100–200 ms) event-related oddball response in EEG and MEG (and as enhanced blood oxygenation signal in fMRI), which surpasses that of a similar pseudoword in amplitude, provided that (1) word and pseudoword stimuli are matched acoustically and phonologically and (2) deviant-standard contrasts in word and pseudoword conditions are identical (Endrass, Mohr, & Pulvermüller, 2004; Korpilahti, Krause, Holopainen, & Lang, 2001; Pulvermüller et al., 2001; Shtyrov & Pulvermüller, 2002; Sittiprapaporn, Chindaduangratn, Tervaniemi, & Khotchabhakdi, 2003; Shtyrov, Pihko, & Pulvermüller, 2005; Shtyrov, Osswald, & Pulvermüller, 2008). Importantly, the relative size of word vs. pseudoword activation is different under attended conditions, where pseudowords produce an enhanced response, usually at a later latency (in the N400 range), at least in the context of word-oriented tasks such as lexical decision (e.g., Friedrich, Eulitz, & Lahiri, 2006). Such a pseudoword-driven enhancement of the N400, often seen in a wider, sentential context (e.g., Federmeier, Segal, Lombrozo, & Kutas, 2000), can be taken as a sign of increased processing load caused by the futile search for an non-existent match in the mental lexicon, as well as re-analysis and repair of ill-shaped linguistic input (cf. Kutas & Hillyard, 1980; Lau, Phillips, & Poeppel. 2008). This is different from the passive conditions necessary for the oddball response enhancement which is linked to automatic activation of word memory traces. Thus the two responses likely reflect different processing steps: early automatic and late attention-controlled stages in word processing (MacGregor, Pulvermüller, van Casteren, & Shtyrov, 2012; Shtyrov, 2010).

To investigate this divergence more closely, attend and non-attend conditions were compared directly in an oddball presentation using the same word and pseudoword stimuli, while manipulating subjects’ attention to the auditory input. This research indicated that, under explicit instructions to listen to spoken stimuli or in a speech-oriented behavioural task, oddball responses to senseless pseudowords are larger than those to meaningful words whereas the reverse – larger word than pseudoword response – is true in non-attend conditions (Garagnani, Shtyrov, & Pulvermüller, 2009; Shtyrov, Kujala, & Pulvermüller, 2010a). Enhanced word activation was interpreted as an automatic activation of long-term memory traces (not present for pseudowords), which can occur automatically in non-attend designs due to the robustness of words’ neural representations (Pulvermüller & Shtyrov, 2006; Shtyrov, 2010). At the same time, most psycholinguistic theories also predict activation for lexical neighbours of words and pseudowords (Marslen-Wilson, 1987). This additional activation quickly becomes extinct when there are no attentional resources available, but, in attend conditions, it is allowed to develop, particularly for pseudoword stimuli (for which lexical selection of a single entry – that can suppress neighbouring traces – cannot be achieved), leading to an increased brain response (Shtyrov, 2010). In addition to electrophysiological investigations, this proposal received clear support and mechanistic explanation from a neurobiologically-based computational model of word memory traces and attention processes in the brain (Garagnani, Wennekers, & Pulvermüller, 2008).

In sum, these differential responses can be interpreted as reflecting activation of word-specific memory traces that is enhanced or suppressed depending on attention allocation to stimulus input. They could therefore be used as a tool to trace lexical processes in the brain and their interaction with attention. However, as the previous studies relied on experimental instructions to attend or ignore the stimuli, a technique for automatic attention modulation (not dependent on explicit instruction) is still necessary to forego the need for an explicit active task, if subjects that are less able to follow such a task are to be tested. Such a technique is proposed and tested in the current study.

It has been long known that unexpected sounds that strongly differ from their environment cause an automatic attention switch, which is what we suggest to use here to allocate attention to linguistic input without an overt instruction. Our suggested design is therefore based on presenting the subjects with a variety of non-linguistic stimuli (tones), sometimes replaced by spoken items, which, due to their marked acoustic differences from the tone stimuli, are bound to draw attention automatically. Such an automatic shift of attention is known to be reflected in brain activity in the form of the so-called P3a deflection (also known as ‘novelty P3’) in electro- or magnetoencephalogram, originating from stimulus-driven attention mechanisms (Friedman, Cycowicz, & Gaeta, 2001; Polich, 2007). The presence or absence of the P3a deflection could therefore be used to probe, on the one hand, stimulus salience in terms of automatic attention shifts, and on the other hand – and more importantly – the nervous system’s ability to process such a novel auditory event.

Still, once the putative attention shift has occurred, how could one test whether the linguistic stimuli are processed as such, not just as a striking acoustic deviance? Here, our previous studies mentioned above make a clear prediction: under conditions when attention is allocated to speech, a rare meaningless pseudoword elicits a larger negative-going^1^ response than a meaningful word as early as 100–200 ms, a phenomenon that is hypothesised to reflect the intensified processes of lexical search (for a review, see Shtyrov, 2010). This divergence is also predicted by the already mentioned neural model of word memory traces realised as strongly linked cell assemblies distributed across auditory perceptual and motor articulatory cortices that become active when presented with linguistic perceptual input (Garagnani et al., 2008). A similar pattern, albeit with a later latency (>250 ms), is seen in active N400 studies that involve, for example, a lexical decision task on word/pseudoword stimuli (Friedrich et al., 2006). Therefore, the presence of such a lexical distinction in the brain response pattern could indicate language-specific processing of stimuli, not merely acoustic deviance aspects.

Obviously, the moment of word/pseudoword divergence must be distinct from the point of speech/non-speech divergence, to allow for attention shifts to occur, on the one hand, and to disentangle the initial effect of auditory novelty from the lexical effect of word-pseudoword contrast, on the other hand. We therefore suggest a stimulation design, in which the attention-modulating speech/non-speech contrast occurs in the very beginning of the stimulus, whereas the lexical contrast is postponed until the very end, to the last phoneme of the speech stimuli which can render the whole stimulus to be a meaningful word or a meaningless pseudoword.

In sum, our suggested paradigm amounts to presenting the subjects with a majority of non-speech stimuli, occasionally replaced (in a modification of an oddball design) with spoken items. Spoken items, in turn, consist of words and pseudowords that only become distinct in the very end, at their last phoneme. We predict: (i) a P3a deflection specific to speech stimuli, at latencies of ∼250 ms relative to the stimulus onset, and (ii) a larger pseudoword than word response at 100–200 ms relative to the onset of the last phoneme. Such effects could be used to probe, on the one hand, the neural function of automatic auditory attention, and, on the other hand, the presence of neural processing of lexical information in the incoming speech. Additionally, (iii) all infrequent stimuli could elicit a mismatch negativity (MMN) response, indicative of automatic auditory discrimination (Näätänen et al., 2007). Aimed in principle at assessing language function in subjects/patients who are not able to cooperate with a more active experimental task, this novel paradigm is for the first time tested here, using high-density multi-channel MEG (generally considered as the most patient-friendly brain imaging technique), in healthy volunteers.

## 2 Methods

### 2.1 Subjects

Twenty healthy right-handed (handedness assessed according to Oldfield (1971)) native English-speaking volunteers (age 19–43, mean age 29.8, 5 females) with normal hearing and no record of neurological diseases were presented with non-speech tones and spoken English language stimuli. All subjects were paid for their participation. Ethics permission for the experiments was granted by the Cambridgeshire Local Research Ethics Committee (LREC, Cambridge, UK).

### 2.2 Stimuli

**Fig. 1:**
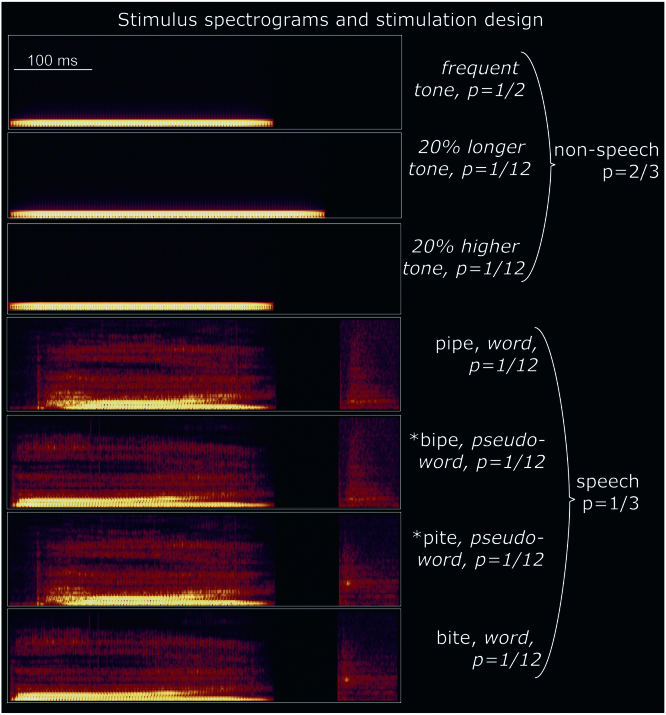
Spectrograms of experimental stimuli. The majority of stimuli (2/3) were non-speech, which guaranteed involuntary switch of auditory attention to the infrequent (1/3) spoken stimuli. These, in turn, could only be disambiguated as words or pseudowords only in their end, at the last phoneme. All spoken stimuli were closely matched acoustically and counterbalanced in their phonetic make-up.

For stimulus presentation, we adapted the so-called ‘optimum’ or ‘multi-feature’ version of the oddball paradigm (Kujala, Tervaniemi, & Schroger, 2007; Näätänen, Pakarinen, Rinne, & Takegata, 2004), which, unlike traditional oddball designs, can accommodate multiple types of rare (‘deviant’) sounds in order to use experimental time more efficiently. In this design, every second stimulus is recommended to be the frequent (‘standard’) sound, whereas the remaining stimulus locations are randomly distributed between the different types of deviants. Our choice of stimuli was determined by the requirements to (1) have an overall majority of non-speech stimuli, (2) ensure the linguistic word and pseudoword spoken stimuli only differ in their end, and (3) balance acoustic and phonetic/phonological contrasts across spoken stimuli to control for low-level perceptual effects. This led to the following stimulation set-up (see also Fig. 1):

1/2 of all stimuli were frequent (standard) 330-ms tones composed of a 275 Hz sine-wave combined, in equal proportion, with its two first harmonics (i.e., 550 and 875 Hz, similar to spoken language, where the main, so-called “fundamental” frequency is complemented by further formants). This sound linearly increased in amplitude over the first 40 ms to reach 50 dB above hearing threshold, and similarly faded out in the last 40 ms, to avoid abrupt onset and offset. A further 1/6 of the stimuli were two non-speech deviants: a 20% longer duration deviant and a 20% higher-pitch frequency deviant, distributed equiprobably. Finally, a minority of stimuli were spoken items: two word (*bite, pipe*) and two pseudoword (^⁎^*bipe*, ^⁎^*pite*^2^) deviants, each having a probability of 1/12. Note that for the *bi-*starting stimuli, a [*t*]-ending results in a word, while the final [*p*]-sound leads to a pseudoword. The opposite is true for the *pi-*starting stimuli. Consequently, acoustic-phonetic features are fully counterbalanced with respect to their contribution to the word-pseudoword distinction across the two pairs of spoken stimuli, meaning that any resultant lexicality effects are not subject to acoustic or phonological confounds. Such stimuli are known to generate robust lexical effects in event-related brain responses (Garagnani et al., 2009; Shtyrov & Pulvermüller, 2002; Shtyrov, Nikulin, & Pulvermüller, 2010b).

Furthermore, in stimulus production, physically identical recordings of initial consonant-diphthong CV fragments (*bi-* or *pi-*), obtained from digitally recorded speech of a native British English speaker and matched acoustically, were used in each combination of [*p/t*]-ending items. This design guaranteed that, whilst all spoken items were clearly different from the tones at the onset, they could only be fully identified at their last phoneme ([*t*] vs. [*p*]). These final phonemes were also made identical across the two combinations, using cross-splicing, to remove any associated acoustic confounds. To avoid co-articulation, they were taken from recordings of acoustically similar but not identical items (*hype, hight*). This technique allowed us to control exactly the point in time when the phonetic contrast occurred and, consequently, when each spoken item could be identified. The initial consonant-diphthong parts of all spoken stimuli were equal to the standard tone both in length (330 ms) and in fundamental frequency (275 Hz). Together with their stop-consonant endings, all spoken stimuli were 485 ms in duration. The word-pseudoword divergence point was at 410 ms following an 80-ms silent closure typical of English stop-consonants and providing (1) an ideal place for cross-splicing the stop-consonants across stimuli for their optimisation, and (2) a silent interval that can used for baseline-correction of neurophysiological responses related to the divergence point. To complete the matching process, all stimuli had identical peak amplitude. The analysis of the naturally-spoken stimuli and their modifications were implemented in Cool Edit 2000 program (Syntrillium Software Corp., AZ, USA).

Following the recommendations for the optimal multi-feature stimulus sequence (Näätänen et al., 2004), each consecutive group of 12 stimuli contained all 6 deviants randomised in a different order within each group occupying, between them, every second position, the remaining positions being taken by the standard stimuli. A total of ∼1350 stimuli were presented in such a pseudo-random sequence, with a jittered stimulus onset-to-onset interval (stimulus onset asynchrony, SOA) of 840–940 ms (mean 890 ms).

### 2.3 Magnetoencephalographic recording

The subjects were seated in light-weight magnetically-shielded room (IMEDCO AG, Hägendorf, Switzerland) and asked to ignore the auditory stimulation and to watch a self-selected silent video. The stimuli were delivered through non-magnetic ear-pieces connected via plastic tubes to acoustic transducers (Etymotic Research Inc, Elk Grove Village, IL, USA) outside the room. Throughout the experiment, the brain’s magnetic activity was continuously recorded using a 306-channel (102 magnetometer and 204 planar gradiometer sensors^3^) Vectorview MEG system (Elekta Neuromag, Helsinki, Finland) with passband 0.10–330 Hz and 1 KHz sampling rate (i.e., sampling rate was ∼50% higher than required by Nyquist–Shannon–Kotelnikov theorem, effectively meaning the data were oversampled for better anti-aliasing). Four magnetic coils were attached to the head and their position was digitized using the Polhemus Isotrak digital tracker system (Polhemus, Colchester, VT, USA). To assist in off-line reconstruction of the head model, an additional set of points randomly distributed over the scalp was also digitized. During the recording, the position of the magnetic coils was continuously tracked (continuous head position identification, cHPI, taken every 200 ms), providing information on the exact position of the head in the dewar. To control for eye-movement artifacts, horizontal and vertical eye movements were recorded using electrooculogram (EOG) electrodes.

### 2.4 Data analysis

Raw MEG recordings were pre-processed using the temporal extension of the signal source separation (tSSS) technique (Taulu & Simola, 2006; Taulu, Kajola, & Simola, 2004), as implemented in MaxFilter 2.0 software (Elekta Neuromag), in order to remove externally-generated noise from the data. Furthermore, we employed the same software to correct for head movements within a recording block, and to correct for different head positions across sessions/participants by re-aligning the data to a common device space (i.e., moving the origin of the sphere fitted to the participant’s scalp surface to the origin of the helmet, and rotating such that the head coordinate system, defined by the nasion and two pre-auricular fiducial markers, was aligned to the device coordinate system). These procedures re-calculate MEG data as if the subject’s head continuously remained in the centre of the measurement device without any motion. Notch filters served to remove contributions from line noise (50 Hz and harmonics), and the high frequency activity generated by the HPI coils (bursts of 293 Hz, 307 Hz, 314 Hz and 321 Hz). Finally, to reduce file sizes and optimise processing times, all data-sets were down-sampled by a factor of four, to 250 Hz sampling rate (with anti-aliasing lowpass filter of 111 Hz employed in down-sampling procedure).

Epochs were then generated from −50 ms to 1000 ms after the sound onset, and averaged to create event-related fields (ERFs) for the six conditions (standard tone, frequency *pipe*, ⁎*pite*, *bipe and bite deviants). Duration deviants (serving as fillers to increase the number of non-speech stimuli) were not analysed as such since they diverged from the standards only at the offset and thus were not directly comparable with the other deviant sounds, which deviated immediately at the onset. Baseline correction was applied by subtracting the averaging average amplitude in the pre-stimulus interval of −50 ms–0 ms from every ERF sample, in each channel separately. Epochs containing artifacts (EOG signal exceeding 150 μV at either of two bipolar eye-movement electrodes or with field-intensity variation exceeding 3000 femtotesla per centimeter, fT/cm) were excluded from averaging. The recordings contained on average 82 accepted responses per deviant stimulus. The analysis proceeded independently on the magnetometer and gradiometer data. For gradiometers, root mean squared (RMS) values were calculated for planar gradiometer pairs at each spatial location (204 gradiometers are grouped into 102 pairs, in which 2 sensors are orthogonal to each other), giving a scalar value representing field gradient magnitude at that location. RMS is computed as a square root of the sum of squared amplitudes recorded in the two sensors.

Significant differences between the standard and deviant tones, as well as between words and pseudowords were identified using a 3-dimensional sensor x time statistical parametric maps using SPM5 software (FIL, London, UK). To this end, 3D (2D sensor x 1D time) images were computed for each participant’s averaged MEG data by projecting the sensor positions onto a 2D grid of 32×32 pixels, and tiling across a third dimension corresponding to each time sample of 4 ms (see also Fig. 3). The data for each voxel within these images were fit by a General Linear Model that included all condition and subject effects. Several linear contrasts of the resulting parameter estimates were estimated across conditions, using a single pooled error whose nonsphericity was estimated by restricted maximum likelihood estimation (Friston et al., 2002). Each contrast resulted in a statistical parametric map (SPM), in which clusters of contiguous suprathreshold voxels (using threshold corresponding to *p*<0.001 uncorrected) were corrected for spatial extent using random field theory (Worsley, Taylor, Tomaiuolo, & Lerch, 2004). Final estimated smoothness was approximately 13 mm in-plane and 21 ms in time for gradiometer RMS SPMs, and 23 mm in-plane and 28 ms in time for magnetometer SPMs.

Our main predictions are based on the amplitude changes in the MEG signal, which could be located in space and time using ERFs as recorded by MEG sensors. Further to this, in an attempt to delineate possible neural origins of ERF effects, their cortical sources were estimated using L2 minimum-norm current estimates (L2 MNE). The minimum-norm method provides a solution to the inverse problem of localising neural activity in the brain from its external recordings by revealing the unique constellation of active neuronal current elements that models the recorded magnetic field distribution with the smallest amount of overall activity (Hämäläinen & Ilmoniemi, 1984; Hämäläinen & Ilmoniemi, 1994; Ilmoniemi, 1993). The L2 MNE does this by determining the combination of sources that have a minimum total power (calculated as an integral of squared current amplitudes).

MNE solutions were calculated for grand-average data, which has a benefit of substantially reduced noise and therefore an improved signal-to-noise ratio (SNR) which MNE solutions are highly sensitive to. A three-compartment (skin-skull-brain) boundary element model (BEM) was used for computing MNE solutions. Cortical grey matter surface was triangularised (∼8000 triangles, i.e., ∼4000 vertices) using an MRI image of a standardised brain (Montreal Neurological Institute), as individual subjects’ MRIs were not available. The currents were restricted to the grey matter with a requirement of normal orientation of current dipoles to the surface (i.e., a single dipole at each vertex). CURRY 6 software (Compumedics Neuroscan, Hamburg, Germany) was used for these procedures. The drawback of such a grand-average source approach is the lack of individual source statistics; in this case, however, a solid statistical support was provided by the signal-level analysis whereas source solutions only have illustratory purposes.

### 2.5 Sleep-deprived subjects

Finally, to test potential applicability of our paradigm to neurocognitive deficits, we repeated this experiment in 7 healthy volunteers who were sleep-deprived for a minimum of 24 h prior to MEG recording. Sleep deprivation is often an associated feature, as well as a possible cause, of psychosis (Gove, 1970; Rimon, Fujita, & Takahata, 1986), which provides an opportunity to model a psychotic condition in healthy individuals by depriving them of normal night-time sleep (Kahn-Greene, Killgore, Kamimori, Balkin, & Killgore, 2007). Given the small number of subjects, their ERF data are provided here for demonstration only; no statistical or source analyses were possible at this stage.

## 3 Results

**Fig. 2:**
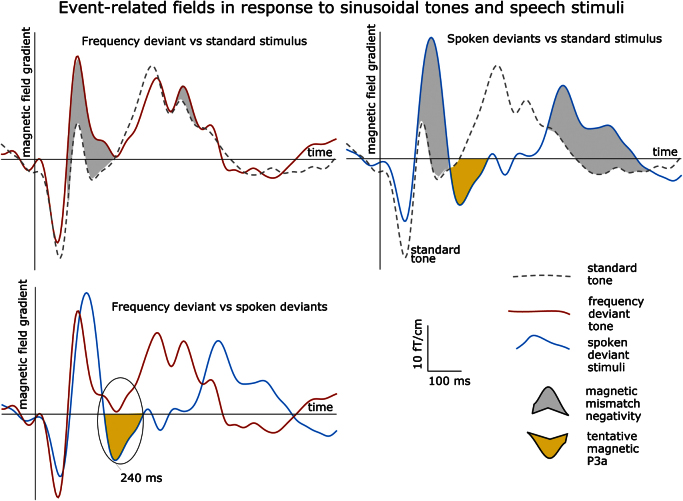
MEG responses (grand average event-related fields) elicited by different types of stimuli. The plot displays time-amplitude dynamics of magnetic field gradients over the left hemisphere at the sensor location with maximum response magnitude (0242): Spoken vs. Frequency deviant vs. Standard tone. Note the MMN-like difference between responses to all deviant and standard stimuli, and the P3a-like ERF deflection present for speech stimuli only.

**Fig. 3:**
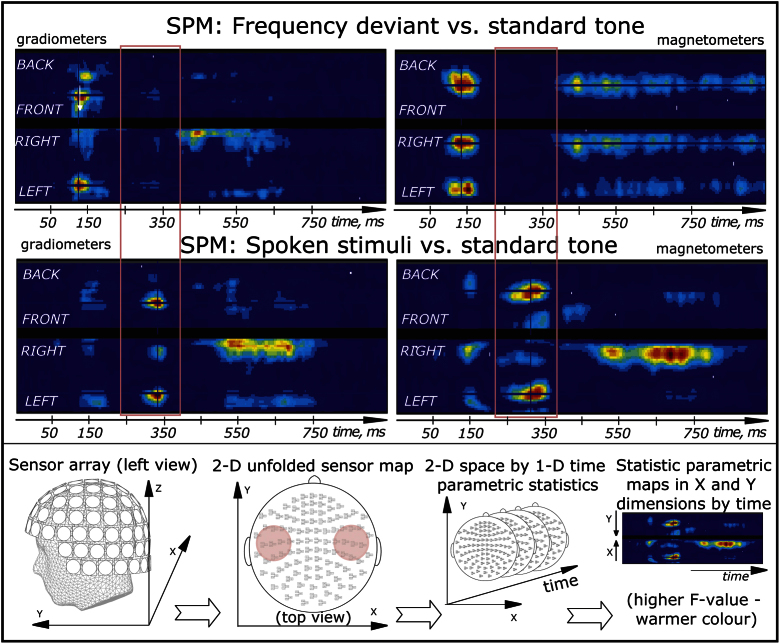
Statistic parametric mapping (SPM) of MEG activity in sensor space and time. (*top*) Typical mismatch response for frequency deviant vs. standard tone. Significant bilateral temporal effects of bi-phasic nature in both gradiometers and magnetometers. (*middle*) For spoken deviants, in addition to the mismatch response with similarly bi-phasic pattern, a P3-like deflection became significant at 190–370 ms across the two sensor types (highlighted in pink). SPMs were corrected for spatial extent in accordance with random field theory. Only significant effects surpassing statistical thresholds (*p*<0.001 uncorrected for height, *p*<0.05 corrected for extent) are shown. (*bottom*) Illustration of SPM sensor space by time statistical mapping approach for MEG: ERF responses from all channels are represented in 2 dimensions in space (*X*, *Y*) and 1 in time. These 3-dimensional images are subjected to statisitical analysis producing statistical parametric maps identifying significant effects in time and in sensor space (see Methods). Highlighted in pink on the 2-dimensional channel layout plot are the areas above temporal lobes where the observed effects were maximal.

The recordings were carried out successfully, and all stimuli elicited magnetic auditory evoked responses (see Fig. 2). The differences between frequent standard and infrequent deviant stimuli were assessed using the 3D SPM analysis (Fig. 3).

For the frequency deviant, we found significant effects (*p*<0.001 uncorrected for height, *p*<0.05 corrected for extent) of frequency deviants that were maximal at a number of points. This mismatch-negativity-like effect (larger deviant than standard ERF) peaked at ∼140 ms after the onset; there also appeared to be a second phase of this response with a maximum at ∼460 ms. The difference between the frequency deviant and the standard response surpassed the significance threshold at 65–186 ms and 387–960 ms in magnetometers and 100–194 ms and 396–666 ms in gradiometers (see Fig. 3 for statistical parametric maps). No obvious P3-like deflection was found for the frequency deviant (Figs. 2 and 3).

Spoken deviant stimuli (pooled together) elicited ERFs that similarly diverged from the standard tone with the earliest peak approximately at 160 ms. Their mean ERF also had a second peak at ∼540 ms (see Fig. 2), which was unsurprisingly later than that of the frequency deviant given the stop-consonant plosion release in the end of all spoken items. These differences between the spoken deviants and the standard reached significance, according to SPMs, at 120–200 ms and 470–800 ms in gradiometers and 120–190 ms and 390–910 ms in magnetometers (see Fig. 3). However, in addition to the bi-phasic mismatch response that generally followed the same pattern as the frequency deviant, spoken stimuli caused an additional deflection of the opposite polarity which peaked at 240 ms (Fig. 2) and proved to be significantly different from the standard tone both in magnetometers (at 190–360 ms) and in gradiometers (230–370 ms). This deflection (which was also significant, with small variations in time, in all 4 individual spoken conditions) seems to correspond best to the P3a wave known from the earlier ERP research. As noted above, no corresponding deflection could be located for the frequency deviant.

**Fig. 4:**
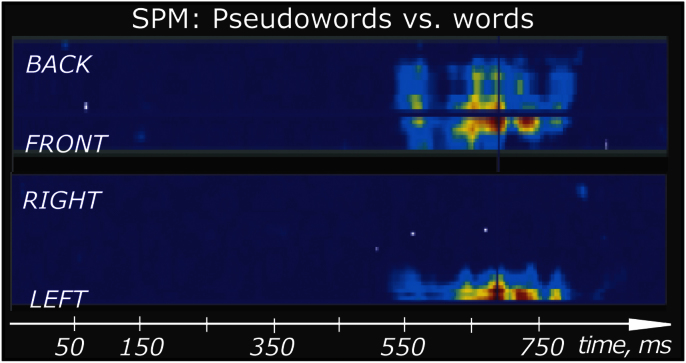
Statistic parametric mapping (SPM) of word-pseudoword differences in MEG activity plotted in sensor space and time. Significant word-pseudoword differences were found after the divergence in left frontal and temporal channels, predominantly in gradiometers. Only significant effects surpassing statistical thresholds (*p*<0.001 uncorrected for height, *p*<0.05 corrected for extent) are shown.

**Fig. 5:**
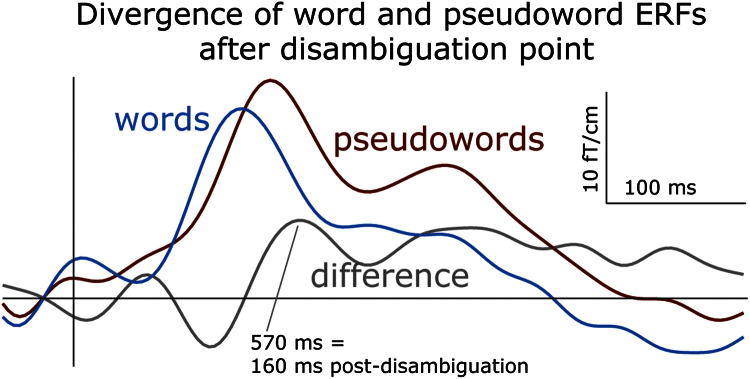
MEG responses (grand average event-related fields) elicited by word and pseudoword stimuli. The plot displays time-amplitude dynamics of magnetic field gradients over the left hemisphere at the sensor with maximum response magnitude (0242), time locked to the divergence point (410 ms post-onset), when the stimuli could be disambiguated and uniquely identified. Note the pseudoword-word difference that peaks at 160 ms after the disambiguation point (570 ms post-onset) and is sustained at later latencies.

Finally, we compared the activation between the rare deviant words and pseudowords. Their activation significantly diverged at 546–855 ms (this effect was seen almost exclusively in the gradiometers, Fig. 4). The peak of this difference, which clearly showed larger pseudoword than word activity, occurred in left temporal channels at 570 ms after the sound onset (Fig. 5), which corresponds to 160 ms after the divergence point (the onset of the last consonant when words and pseudowords could be uniquely identified).

**Fig. 6:**
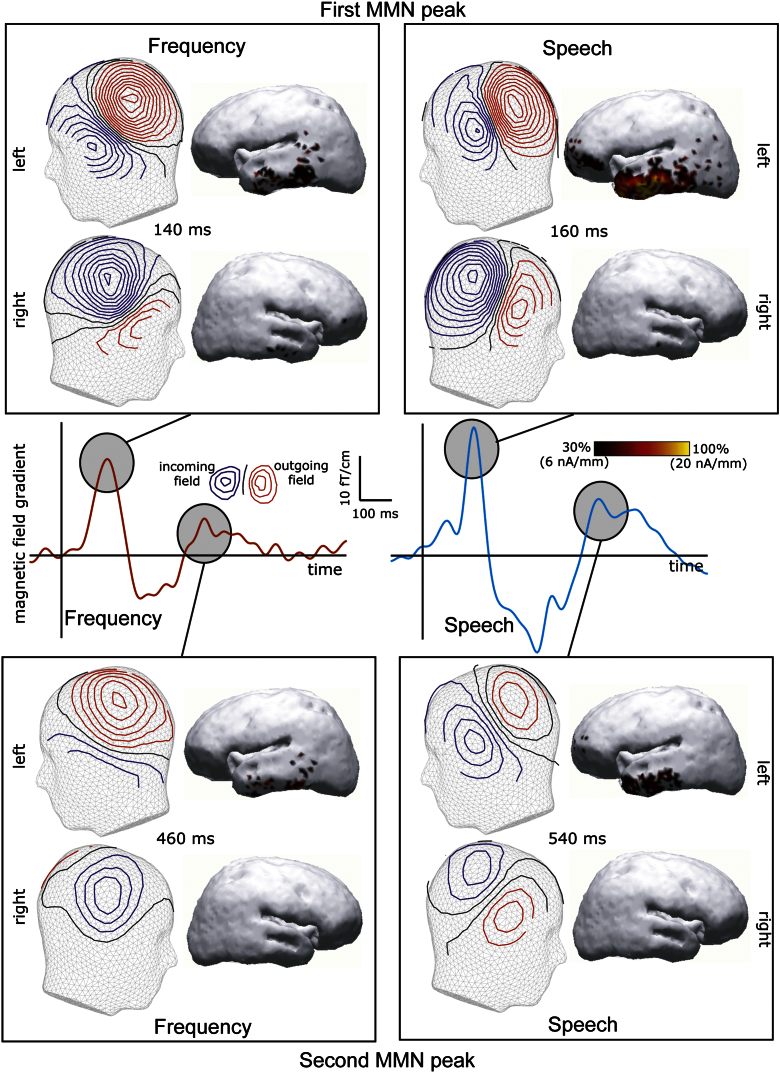
Time course and cortical topography of the mismatch negativity response elicited by frequency and speech deviants: time-amplitude dynamics of magnetic field gradients (deviant minus standard subtraction) at the sensor 0242 which exibited maximum response magnitude (middle), grand-mean MEG field topographies and distributed source models (L2 minimum-norm current estimates) for the early and late MMN deflection in the two hemispheres. ERF and source maps shown are taken at the response peak latency. For scaling, the maximum source activation at this time is taken as 100%.

**Fig. 7:**
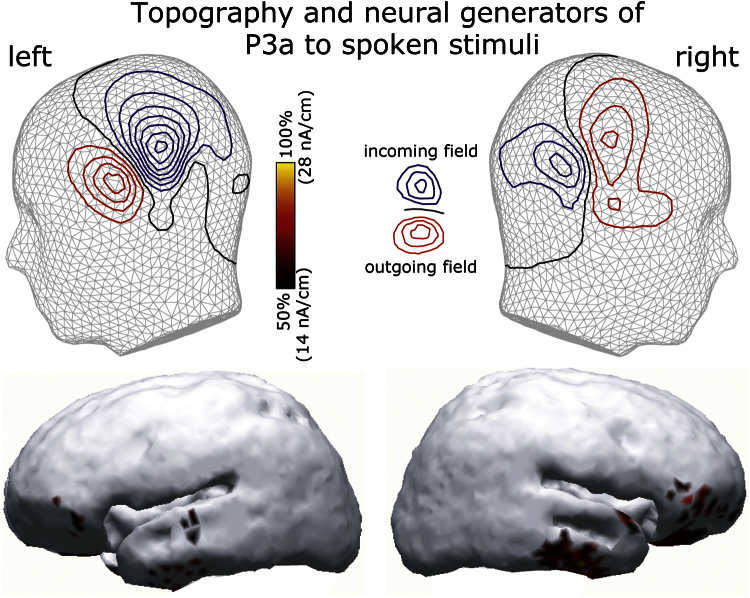
Cortical topography of P3-like deflection in response to spoken deviants among non-speech stimuli: grand-mean MEG field topographies and distributed source models (L2 minimum-norm current estimates) in the left and right cerebral hemispheres. ERF and source maps shown are taken at the response peak latency. For scaling, the maximum source activation at this time is taken as 100%.

**Fig. 8:**
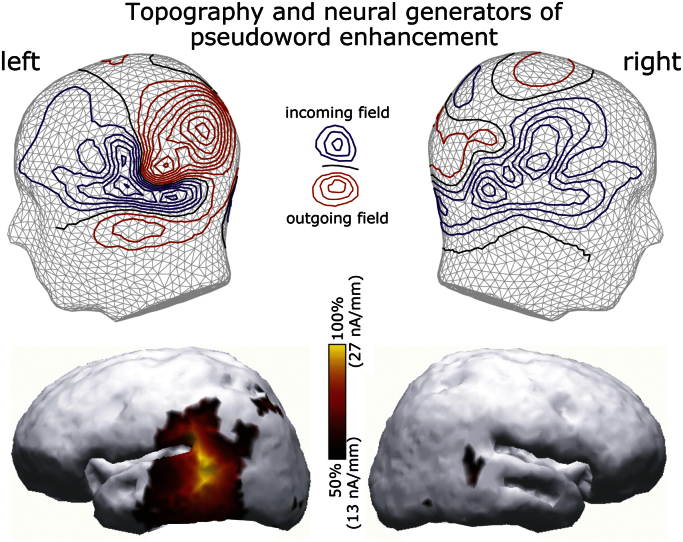
Cortical topography of pseudoword advantage over the word activity: grand-mean MEG field topographies and distributed source models (L2 minimum-norm current estimates) in the left and right cerebral hemispheres computed for a difference (pseudword minus word ERF) response. ERF and source maps shown are taken at the response peak latency. For scaling, the maximum source activation at this time is taken as 100%.

A magnetic MMN-like deflection that uniformly differentiated between infrequent deviants and frequent standard tones (similar for frequency and spoken deviants) has been repeatedly shown to predominantly originate from temporal sources (Alho et al., 1996; Picton, Alain, Otten, Ritter, & Achim, 2000; Shtyrov et al., 2005); the current data confirmed these previous findings (Fig. 6). However, sources of the P3a-effect elicited by rare spoken stimuli and of the larger pseudoword than word activity at 160 ms post-identification have been scarcely investigated. To explore these effects further, we attempted to reconstruct their cortical sources using distributed minimum-norm source estimates. The P3a-like effect appeared to be generated by distributed bilateral neuronal sources in predominantly temporal and inferior-frontal areas (Fig. 7), whilst the attention-caused pseudoword advantage over word stimuli appeared to be predominantly sustained by activity in the left middle and posterior temporal cortex (Fig. 8).

**Fig. 9:**
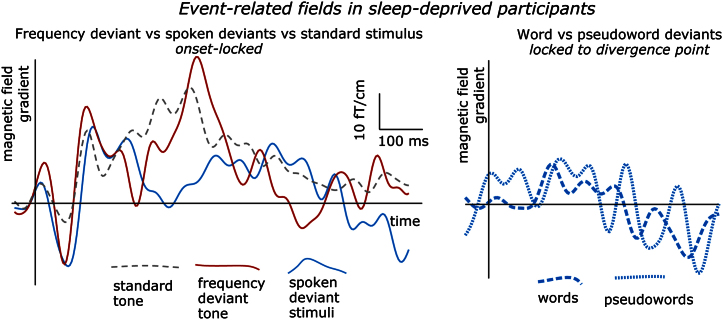
MEG responses (grand average event-related fields) elicited by different types of stimuli in sleep-deprived individuals. The plot displays time-amplitude dynamics of magnetic field gradients over the left hemisphere at the sensor with maximum response magnitude (0242): Spoken vs. Frequency deviant vs. Standard tone from stimulus onset (left) and Word vs. Pseudoword from disambiguation point (right). Note the reduction of MMN-like difference between responses to deviant and standard stimuli in comparison to normal data, and the breakdown of P3a-like ERF deflection for speech stimuli as well as the absence of a defined response after the disambiguation point (cf. Figs. 2 and 5).

In an attempt to test our paradigm in a model pathological neurocognitive state, we also recorded MEG data in small set of sleep-deprived individuals. These results (Fig. 9), albeit not subject to statitistical analysis, show a stark contrast with our main testing group. Whilst a residual MMN pattern could be observed, visual inspection indicates neither P3a-like activity nor word-pseudoword differences after the disambiguation point.

## 4 Discussion

In this study, we have suggested and tested a novel paradigm which, in the absence of focussed attention on the auditory input, can shift attention to the spoken stimulus thereby affecting its lexical processing. Lexical differences (word-pseudoword distinction) were postponed to the very end of the stimuli to disentangle their effects from those of the earlier acoustic deviation. We registered the brain’s activity elicited by non-speech and speech stimuli in this paradigm using high-density whole-head MEG equipment and analysed resulting event-related fields. Three main results are as follows:

1. Non-speech (frequency) and speech deviants that diverge from the standard non-speech tone at the sound onset elicit a bi-phasic response showing increased amplitude over that to the standard tone.
2. In addition, rare speech sounds elicit a P3a-like deflection that has the opposite polarity to the main mismatch peaks and reaches maximum at ∼240 ms after the stimulus onset.
3. At ∼160 ms after the spoken stimuli can be uniquely identified (at their last phoneme), their responses diverge showing larger neural activation for meaningless pseudowords as opposed to meaningful words.

Below, we will briefly consider our findings in more detail:

### 4.1 Deviant-standard differences

Deviant stimuli, both non-speech and spoken, produced a response that was markedly different from that to the standard tone: a prominent peak at ∼150 ms, followed by a second wave which peaked somewhat earlier (460 ms) for non-speech and later (540 ms) for speech sounds. This amplitude increase for rare auditory stimuli as opposed to more frequent ones is best described as the magnetic equivalent for the mismatch negativity (MMN) response, first described by Näätänen, Gaillard, and Mäntysalo (1978). MMN is an evoked brain response elicited by rare unexpected deviant acoustic stimuli occasionally presented in a sequence of frequent standard stimuli (Alho, 1995; Näätänen, 1995). Importantly, MMN (and its magnetic counterpart, MMNm) can be elicited in the absence of the subject’s attention to the auditory input (Schröger, 1996; Tiitinen, May, Reinikainen, & Näätänen, 1994) whilst its magnitude and latency are dependent on the acoustic discrepancy between standard and deviant stimuli. It is therefore thought to reflect the brain’s automatic discrimination of changes in the auditory sensory input and thus to be an indicator of automatic cerebral processing of acoustic events (Näätänen, 1995). The current paradigm is different from the ‘classical’ MMN design, in which stimulus order is fully random, and the standards constitute the absolute majority (e.g., >80%); here, standard stimuli take up 50% and occupy every second position. Still, this ‘multi-feature’ paradigm appears to generate results very similar to classical MMN designs (Kujala et al., 2007; Näätänen et al., 2004). Appearance of the MMN(m) in non-attend electrophysiological recordings is usually taken as a sign of functional (if not intact) neural mechanisms of auditory discrimination, which can be used as an indicator of the brain’s neurological status, e.g., in brain-damaged patients (Fischer, Morlet, & Giard 2000). From this point, the ability of the current paradigm to evoke an MMNm could be beneficial for using it to assess basic auditory functions, in addition to our originally set goals concerned with attentional and linguistic processes.

Here, we are reporting a bi-phasic MMN response. Although most previous work concentrated on the first MMN peak that normally occurs before 200 ms, it appears that the second wave is often present even in those experiments that do not discuss it explicitly (see e.g., Näätänen et al., 2004); we have certainly observed this bi-phasic nature in our previous studies (Pulvermüller, Shtyrov, Hasting, & Carlyon, 2008; Shtyrov et al., 2010a). Consistent with the current data, a suggestion has been made to separate the MMN into early and late components (Korpilahti et al., 2001). Some previous research interpreted the early peak as a reflection of fully automatic attention-independent processing, whilst the later activation is more subject to top-down attention-dependent control, at least for linguistic stimuli (Shtyrov, 2010).

### 4.2 P3a to spoken stimuli

Although the MMNm here is elicited by spoken and frequency deviants alike, speech-evoked ERFs demonstrate a more complex profile: in addition to the MMNm peaks, they generate another intermediate peak of the opposite polarity. Although one cannot speak of ‘positive’ and ‘negative’ peaks in relation to MEG in the same way as when presenting EEG data, it is important to point out that this deflection, which reached its maximum at 240 ms in temporal channels, was reversed in polarity as compared with the MMNm, which makes it a likely magnetic counterpart of a positive event-related potential. Its timing clearly suggests that this is a P3a-like response. This interpretation is further supported by the fact that it was only present for the rare speech stimuli that were least expected among the majority of non-speech tones of three types. Novel non-speech stimuli have been known to elicit a P3a component, in addition to the MMN, even when they are not attended (Paavilainen, Karlsson, Reinikainen, & Näätänen, 1989). Whilst the MMN has been linked to detection of a distracting sound, the P3a event-related response has been interpreted as associated with the subsequent involuntary orienting of attention to the novel sound and, more generally, with the evaluation of novel events for subsequent behavioural action (Escera, Yago, & Alho, 2001; Friedman et al., 2001). P3a has been found both in EEG and MEG and in both auditory and visual modalities (Alho et al., 1998; Jeon & Polich, 2001). Both temporal and frontal sources were suggested to take part in P3a generation (although to a different extent in visual and auditory modalities, Alho et al., 1998; Polich, 2007), which is fully supported by the current results. It has been previously suggested to be of clinical utility for the assessment of cognitive disorders (Polich, 2004). The latter is in line with the aims of the current study, where we attempt to estimate the nervous system’s ability to re-orient its attention automatically to such ecologically important and uniquely human information input as spoken language. The important difference between the current study and previous research is that we have used speech stimuli for P3a elicitation in a passive task whereas the majority of previous research utilised non-linguistic novel stimuli and frequently required active discrimination tasks. Clearly, automatic attention shifts to speech successfully take place in the current setting, where healthy volunteers are under investigation; testing this paradigm with neurological patients whose speech function is impaired (e.g., in aphasia) would be an important next step.

One potential limitation of the results we present is that there is no unattended speech condition to use as a baseline against which the P3a (and other effects) could be tested. This is precluded by the design of the current study which intentionally avoids a fully non-attend condition, concentrating instead on the effects of involuntary attention shift to spoken stimuli as the main target. However, there is solid body of evidence available from previous research using words and pseudowords in fully non-attend designs. Those studies, as reviewed in the Introduction, consistently reported a lexical response enhancement for words as opposed to pseudowords in the absence of attention, as well as the reversal of this effect when attention is paid to the stimuli. More importantly in the context of this discussion, none of the numerous previous studies using passive non-attend designs with spoken words found a P3a effect. At the same time, such an effect was reported in a direct comparison of attend and non-attend conditions as arising from voluntary attention allocation to speech, achieved through an overt experimental instruction and stimulus-related target detection task (Shtyrov et al., 2010a). Thus, the current data exhibit the same pattern as those with an overt instruction to attend to spoken material, which provide a direct support for our interpretation of the current effects as stemming from an attention shift. The important addition to the previous literature is that we control attention allocation here without an overt instruction or behavioural task by causing it to occur involuntarily.

Another potential criticism that can be made is that we compare acoustically very different stimuli – speech and tones – and the pattern of P3a responses to them may not be the same, with a possibly reduced P3a effect for tones related to their acoustic properties. Also, the SNR for tone stimuli could potentially be lower since they were fewer in number. However, on the one hand, also separately analysed individual speech stimuli could be seen here as evoking P3a. On the other hand, and more importantly, previous studies have established a solid P3 to non-speech stimuli when they are attended to (or when attention is forced to them by a strong deviance), and its absence in passively presented unattended tonal contrasts (Alho et al., 1998; Escera et al., 2001; Näätänen, 1992; Paavilainen et al., 1989). This is in line with our interpretation of the current design as not drawing attention to the tones, thus preventing the appearance of a P3a, which can, however, be seen for the spoken items. Future studies could use further modifications of our design in combination with additional control conditions in order to elucidate the effects we report in more detail.

More generally, such an attention shift effect as reported here need not be limited to speech stimuli. We would predict that any strongly deviant stimulus – not necessarily speech – should attract attention, which would in turn generate a P3 response. However, the critical advantage of using speech is that, as a result of attention allocation, we also obtain a specific pattern of lexical effects which will be discussed below.

### 4.3 Pseudoword-word difference

Finally, when analysing word and pseudoword responses separately, we found clear differences at >100 ms after these items could be identified: an elevated response to pseudowords. Acoustic/phonological features of the stimuli were fully balanced: both words and pseudowords equally included the initial segments *bi-* and *pi-* and final stop consonants *p* and *t*. Moreover, these segments were physically identical, but combined in a different order in order to create meaningful words or meaningless pseudowords. This makes a purely acoustic explanation rather unlikely, and suggests that the differences are caused by the linguistic features of the stimuli. Previous research has shown an increased response to pseudoword over word items, which is usually most apparent in a later time range (e.g., N400 as in Federmeier et al., 2000 or Carreiras, Vergara, & Barber, 2005) and could be linked to enhanced search in the mental lexicon for a representation that does not exist. Earlier effects of lexicality, including a range of word-pseudoword differences at latencies before 200 ms have also been found (for a review, see e.g., Shtyrov & Pulvermüller, 2007b), pointing to rapid access to the mental lexicon which has been long postulated by behavioural psycholinguistic studies (Marslen-Wilson, 1987, 1990).

Previous research using passive oddball paradigms has, however, consistently indicated a higher response to words than pseudowords (e.g., Korpilahti et al., 2001; Shtyrov & Pulvermüller, 2002), which is the opposite of the pattern observed here. The main difference between the current study and previous research is that here spoken items constitute a minority of stimuli, interspersed among sinusoidal standards and frequency and duration deviants, whereas in all previous studies words and pseudowords were presented among other spoken items. This specific feature of the current design, as we argued above, must have caused an automatic shift of auditory attention to them, which was evident in the generation of a magnetic P3a visible for spoken stimuli (but not others). This in turn means that, by the time the disambiguation point arrived in the end of speech stimuli, re-orienting of auditory attention had already taken place, and they were attended to. In conditions where spoken stimuli are attended, pseudoword-elicited activation is known to dominate the event-related response; this is true both in N400 (Friedrich et al., 2006) and in oddball MMN-type designs (Garagnani et al., 2009). More specifically, it was found that while early word responses are largely unchanged by attentional modulation, allocating attention to pseudoword input increases its corresponding event-related response amplitude in EEG and MEG (Garagnani et al., 2009; Shtyrov et al., 2010a). This enhanced auditory pseudoword response was also visible in active paradigms that have used fMRI, where it was located in the middle and posterior left temporal cortices (Newman & Twieg, 2001; Hugdahl, Thomsen, Ersland, Rimol, & Niemi, 2003), which is well in line with the configuration of sources we found in the current study.

Although we chiefly speak of lexical processing here, we cannot rule out that some of the contribution to differential brain responses we observe is semantic in nature. In fact, reflections of automatic semantic access at similar early latencies were found in passive oddball designs (Menning et al., 2005; Pulvermüller, Shtyrov, & Ilmoniemi, 2005; Shtyrov & Pulvermüller, 2007a; Shtyrov, Hauk, & Pulvermüller, 2004), which suggests that the word-specific activation potentially includes a semantic element; it may therefore be more appropriate to speak of lexico-semantic access. In either case, the processes under consideration are related to linguistic memory trace activation. Since our paradigm was not specifically aimed at addressing semantic distinctions, we refrain from further speculations on this issue. Future modifications of this paradigm may incorporate semantic distinctions in the same way as lexical ones were implemented here.

Another word of caution must be mentioned regarding the generalisations that can be drawn from using a very limited number of stimuli to the entirety of language. By the virtue of the oddball design, only very few tokens, repeated multiple times, can be used in any one experiment. Whilst this has a number of methodological benefits which we mentioned before, it does pose a question of ecological validity of such repetitive oddball and multi-feature designs. On the one hand, results achieved with a small number of items cannot be refuted per se. On the other hand, the important role in establishing the reality of early lexical effects in such designs was played by replicating these studies with different settings. To date, automatic lexical effects in passive oddball presentation were found using different imaging methods (EEG, MEG, fMRI), languages (e.g., English, Finnish, German, Russian, Thai, Spanish) by different labs using various stimulation set-ups. Most importantly, with careful matching of word and pseudoword stimuli, nearly identical effects could also be found in the absence of any stimulus repetition, by contrasting large groups of unique words and pseudowords (MacGregor et al., 2012). This supports the notion that the early effects we observe here are indeed linked to the lexical processing of the word and pseudoword stimuli which takes place automatically, regardless of the multiple repetition of the same tokens.

What, however, could be the explanation for such a markedly different pseudoword behaviour in attend and non-attend conditions, and, more specifically, for the pseudoword advantage in the former? Whereas it appears that pseudowords, phonological in nature and phonotactically legal, may activate language areas in the brain (Mazoyer et al. 1993; Newman & Twieg, 2001; Petersen, Fox, Snyder, & Raichle, 1990; Posner, Abdullaev, McCandliss, & Sereno, 1996; Price, Wise, & Frackowiak, 1996; Shtyrov et al., 2005, 2008), the absence of long-term memory traces for such items implies that no activation of such traces *per se* is possible. Therefore, in non-attend conditions they produce a reduced response possibly reflecting acoustic and phonological aspects of their processing as well as partial activation of related lexical traces. The latter assumes a path of pseudoword perception compatible with Cohort (and some other) models of speech processing (Marslen-Wilson, 1987). That implies that all lexical neighbours become partially activated as soon as the relevant information is available. However, as additional information arrives, they become progressively excluded until there is only one candidate left (or none for pseudowords). In the context of this study, its design makes sure that this crucial information arrives at the same time in the end of the stimuli. At this time, the traces for lexical neighbours, activated partially and equally for words and pseudowords, already become extinguished as they are ruled out (once the stimulus is identified, its representation is hypothesised to inhibit other lexical neighbours/competitors)*, if there are no additional resources (attention)* to develop and maintain their activity. The initial word activation seems to reach its maximum regardless of the level of attention due to strong links within the network subserving it. In the associative Hebbian logic of memory trace formation (Braitenberg & Schüz, 1992; Hebb, 1949; Pulvermüller, 1999), the strength of synaptic connections between subparts of such networks is dependent on the degree of co-activation between these parts. Thus, frequent use of words should lead to representations robust enough to such an extent that they are activated by the respective stimulus regardless of amount of attention paid to it. Pseudowords as such have no individual representations and thus produce a reduced response in non-attend conditions, as seen repeatedly in previous passive non-attend oddball experiments. However, with more attentional resources becoming available, the early word activation still remains at its maximum, whereas partial but multiple neighbour activations appear to be able to benefit from these additional resources and thus be maintained at a higher level, leading to the pseudoword advantage effect (for review, see Shtyrov, 2010), which we also observed here. In other words, once the specific representation/lexical entry is selected in the case of a meaningful word, its neighbours are likely to be inhibited/suppressed, as posited in the majority of psycholinguistic theories of word recognition. In the attended pseudoword case, however, this selection point is never achieved, and thus competitors may not be fully ruled out. Furthermore, when attention is actively paid to such ill-shaped linguistic stimuli, they may prompt a more elaborate lexical search (and even reanalysis/repair) once the first attempt on automatic mapping of the input to a single entry fails. This leads to activation enhancement for pseudowords, whilst the initial word memory trace activation is robust and stable irrespective of attention and task demands.

These electrophysiolocal distinctions, predicted by a neurocomputational model of word representations in the brain (Garagnani et al., 2008), have been well documented in previous studies, particularly in passive MMN (word>pseudoword) and N400 (pseudoword>word) investigations, as reviewed above. The important difference between previous studies and the current one is that here we do not use an explicit instruction to focus the subjects’ attention to the stimulus stream. This is done by the design in which attention is drawn automatically to spoken input. The current paradigm therefore makes it possible to probe neural processes of automatic lexical access and selection in the absence of an overt instruction or task to carry out linguistic activity.

### 4.4 Effects of sleep deprivation

Lastly, we have pre-tested the performance of this paradigm in a set of sleep-deprived individuals. Sleep deprivation is often associated with psychotic-type pathologies (Gove, 1970; Kahn-Greene et al., 2007; Rimon et al., 1986). It therefore provides a relatively safe and easily-controlled model of psychotic conditions, which are some of potential future applications for the novel paradigm we suggest here, given many reports of attention and language disturbances in psychotic individuals (such as schizophrenia, see e.g., Braff, 1993; Maher, 1991). While a residual MMN response could be seen in the sleep-deprived individuals, we observed a breakdown of the P3a component, indicating deficits in attention allocation processes, and, furthermore, no indication of lexicality effects (if anything, post-disambiguation responses to spoken stimuli are reduced to noise levels; Fig. 9). This suggests a deterioration of automatic lexical search and selection processes as a result of sleep deprivation. As only a small group of subjects was tested, meaning that the results cannot be verified statistically, these preliminary data must be treated with caution. Future studies are therefore necessary to investigate interactions between automatic lexical and attention processes in sleep deprivation and in clinical conditions.

## 5 Conclusions

In sum, we propose a paradigm that can test, in a single short non-invasive procedure: (1) neural discrimination of acoustic information, (2) neural mechanisms of automatic attention allocation and (3) neural correlates of lexical processing. It does not require explicit instructions and needs minimal cooperation from the subject, therefore having a great potential for assessing auditory and linguistic functions in subjects/patients who are unable to cooperate with a more active task. This paradigm has been tested here using a group of healthy volunteers. Future studies are necessary to investigate its potential implementation in clinical populations.

## Footnotes

1 Negative polarity of this response is observed in EEG at midline electrodes (e.g., Shtyrov et al., 2010) when using average, nose or mastoid reference. In MEG, the notion of voltage polarity is absent, but the pattern can be well-observed as a larger field gradient in event-related fields (ERF) over the temporal lobes (Garagnani, Shtyrov, & Pulvermüller, 2009).

2 Here and throughout we use a linguistic convention an marking pseudo-words with an asterisk (^⁎^).

3 Magnetometers record magnetic field as such (and are thus sensitive to remote sources, but susceptible to ambient electromagnetic interference), whilst planar gradiometers record magnetic field gradient over their surface (and thus are more sensitive to nearby generators and less influenced by remote noise sources).

## References

[bib1] Aitchison J. (2002). Words in the mind: an introduction to the mental lexicon.

[bib2] Alho K. (1995). Cerebral generators of mismatch negativity (MMN) and its magnetic counterpart (MMNm) elicited by sound changes. Ear and Hearing.

[bib3] Alho K., Tervaniemi M., Huotilainen M., Lavikainen J., Tiitinen H., Ilmoniemi R.J., Knuutila J., Näätänen R. (1996). Processing of complex sounds in the human auditory cortex as revealed by magnetic brain responses. Psychophysiology.

[bib4] Alho K., Winkler I., Escera C., Huotilainen M., Virtanen J., Jääskelänen I.P., Pekkonen E., Ilmoniemi R.J. (1998). Processing of novel sounds and frequency changes in the human auditory cortex: magnetoencephalographic recordings. Psychophysiology.

[bib5] Baron-Cohen S., Hoekstra R.A., Knickmeyer R., Wheelwright S. (2006). The autism-spectrum quotient (AQ)—adolescent version. Journal of Autism and Developmental Disorders.

[bib6] Benton A.L. (1994). Neuropsychological assessment. Annual Reviews of Psychology.

[bib7] Braff D.L. (1993). Information processing and attention dysfunctions in schizophrenia. Schizophrenia Bulletin.

[bib8] Braitenberg V., Schüz A., Wind J., Chiarelli B., Bichakjian B.H., Nocentini A., Jonker A. (1992). Basic features of cortical connectivity and some considerations on language. Language origin: a multidisciplinary approach.

[bib9] Carreiras M., Vergara M., Barber H. (2005). Early event-related potential effects of syllabic processing during visual word recognition. Journal of Cognitive Neuroscience.

[bib10] Dale A.M., Halgren E. (2001). Spatiotemporal mapping of brain activity by integration of multiple imaging modalities. Current Opinion in Neurobiology.

[bib11] Endrass T., Mohr B., Pulvermüller F. (2004). Enhanced mismatch negativity brain response after binaural word presentation. European Journal of Neuroscience.

[bib12] Escera C., Yago E., Alho K. (2001). Electrical responses reveal the temporal dynamics of brain events during involuntary attention switching. European Journal of Neuroscience.

[bib13] Federmeier K.D., Segal J.B., Lombrozo T., Kutas M. (2000). Brain responses to nouns, verbs and class-ambiguous words in context. Brain.

[bib14] Fischer C., Morlet D., Giard M. (2000). Mismatch negativity and N100 in comatose patients. Audiology and Neurotology.

[bib15] Folstein M.F., Folstein S.E., McHugh P.R. (1975). “Mini-mental state”. A practical method for grading the cognitive state of patients for the clinician. Journal of Psychiatric Research.

[bib16] Friederici A. (2002). Towards a neural basis of auditory sentence processing. Trends in Cognitive Sciences.

[bib17] Friedman D., Cycowicz Y.M., Gaeta H. (2001). The novelty P3: an event-related brain potential (ERP) sign of the brain’s evaluation of novelty. Neuroscience & Biobehavioral Reviews.

[bib18] Friedrich C.K., Eulitz C., Lahiri A. (2006). Not every pseudoword disrupts word recognition: an ERP study. Behavioral and Brain Functions.

[bib19] Friston K.J., Glaser D.E., Henson R.N., Kiebel S., Phillips C., Ashburner J. (2002). Classical and Bayesian inference in neuroimaging: applications. NeuroImage.

[bib21] Garagnani M., Wennekers T., Pulvermüller F. (2008). A neuroanatomically grounded Hebbian-learning model of attention-language interactions in the human brain. European Journal of Neuroscience.

[bib20] Garagnani M., Shtyrov Y., Pulvermüller F. (2009). Effects of attention on what is known and what is not: MEG evidence for functionally discrete memory circuits. Frontiers in Human Neuroscience.

[bib22] Gove W.R. (1970). Sleep deprivation: a cause of psychotic disorganization. American Journal of Science.

[bib23] Hagoort P. (2008). The fractionation of spoken language understanding by measuring electrical and magnetic brain signals. Philosophical Transactions of the Royal Society of London, Series B: Biological Sciences.

[bib24] Hämäläinen, M. S. & Ilmoniemi, R. J. (1984). Interpreting measured magnetic fields of the brain: minimum norm estimates of current distributions. *Helsinki University of Technology, technical report TKK-F-A559*.

[bib25] Hämäläinen M.S., Ilmoniemi R.J. (1994). Interpreting magnetic fields of the brain: minimum norm estimates. Medical and Biological Engineering and Computing.

[bib26] Hebb D.O. (1949). The organization of behavior. A neuropsychological theory.

[bib27] Henson R.N., Price C.J., Rugg M.D., Turner R., Friston K.J. (2002). Detecting latency differences in event-related BOLD responses: application to words versus nonwords and initial versus repeated face presentations. NeuroImage.

[bib28] Hugdahl K., Thomsen T., Ersland L., Rimol L.M., Niemi J. (2003). The effects of attention on speech perception: an fMRI study. Brain and Language.

[bib29] Ilmoniemi R.J. (1993). Models of source currents in the brain. Brain Topography.

[bib30] Jeon Y.W., Polich J. (2001). P3a from a passive visual stimulus task. Clinical Neurophysiology.

[bib31] Kahn-Greene E.T., Killgore D.B., Kamimori G.H., Balkin T.J., Killgore W.D. (2007). The effects of sleep deprivation on symptoms of psychopathology in healthy adults. Sleep Medicine.

[bib32] Korpilahti P., Krause C.M., Holopainen I., Lang A.H. (2001). Early and late mismatch negativity elicited by words and speech-like stimuli in children. Brain and Language.

[bib33] Kujala T., Tervaniemi M., Schroger E. (2007). The mismatch negativity in cognitive and clinical neuroscience: theoretical and methodological considerations. Biological Psychology.

[bib34] Kutas M., Hillyard S.A. (1980). Reading senseless sentences: brain potentials reflect semantic incongruity. Science.

[bib35] Lau E.F., Phillips C., Poeppel. D. (2008). A cortical network for semantics: (de)constructing the N400. Nature Reviews Neuroscience.

[bib36] Laureys S., Owen A.M., Schiff N.D. (2004). Brain function in coma, vegetative state, and related disorders. Lancet Neurology.

[bib37] MacGregor L.J., Pulvermüller F., van Casteren M., Shtyrov Y. (2012). Ultra-rapid access to words in the brain. Nature Communications.

[bib38] Maher B.A., Steinhauer S.R., Gruzelier J.H., Zubin J. (1991). Language and schizophrenia. Handbook of schizophrenia, Vol.5: neuropsychology, psychophysiology and information processing.

[bib39] Marslen-Wilson W.D. (1975). Sentence perception as an interactive parallel process. Science.

[bib40] Marslen-Wilson W., Altmann G. (1990). Activation, competition, and frequency in lexical access. Cognitive models of speech processing: psycholinguistic and computational perspectives.

[bib41] Marslen-Wilson W.D. (1987). Functional parallelism in spoken word-recognition. Cognition.

[bib42] Mazoyer B.M., Tzourio N., Frak V., Syrota A., Murayama N., Levrier O., Salamon G., Dehaene S., Cohen L., Mehler J. (1993). The cortical representation of speech. Journal of Cognitive Neuroscience.

[bib43] Menning H., Zwitserlood P., Schoning S., Hihn H., Bolte J., Dobel C., Mathiak K., Lutkenhoner B. (2005). Pre-attentive detection of syntactic and semantic errors. NeuroReport.

[bib44] Näätänen R. (1992). Attention and brain function.

[bib45] Näätänen R. (1995). The mismatch negativity: a powerful tool for cognitive neuroscience. Ear and Hearing.

[bib46] Näätänen R., Gaillard A.W., Mäntysalo S. (1978). Early selective-attention effect on evoked potential reinterpreted. Acta Psychologica (Amst).

[bib47] Näätänen R., Paavilainen P., Rinne T., Alho K. (2007). The mismatch negativity (MMN) in basic research of central auditory processing: a review. Clinical Neurophysiology.

[bib48] Näätänen R., Pakarinen S., Rinne T., Takegata R. (2004). The mismatch negativity (MMN): towards the optimal paradigm. Clinical Neurophysiology.

[bib49] Newman S.D., Twieg D. (2001). Differences in auditory processing of words and pseudowords: an fMRI study. Human Brain Mapping.

[bib50] Oldfield R.C. (1971). The assessment and analysis of handedness: the Edinburgh inventory. Neuropsychologia.

[bib51] Owen A.M., Coleman M.R. (2008). Functional neuroimaging of the vegetative state. Nature Reviews Neuroscience.

[bib52] Paavilainen P., Karlsson M.L., Reinikainen K., Näätänen R. (1989). Mismatch negativity to change in spatial location of an auditory stimulus. Electroencephalography and Clinical Neurophysiology.

[bib53] Petersen S.E., Fox P.T., Snyder A., Raichle M.E. (1990). Activation of extra-striate and frontal cortical areas by visual words and word-like stimuli. Science.

[bib54] Pettigrew C.M., Murdoch B.M., Chenery H.J., Kei J. (2004). The relationship between the mismatch negativity (MMN) and psycholinguistic models of spoken word processing. Aphasiology.

[bib55] Picton T.W., Alain C., Otten L., Ritter W., Achim A. (2000). Mismatch negativity: different water in the same river. Audiology and Neurotology.

[bib56] Polich J. (2004). Clinical application of the P300 event-related brain potential. Physical Medicine and Rehabilitation Clinics of North America.

[bib57] Polich J. (2007). Updating P300: an integrative theory of P3a and P3b. Clinical Neurophysiology.

[bib58] Posner M.I., Abdullaev Y.G., McCandliss B.D., Sereno S.C., Everatt J. (1996). Anatomy, circuitry and plasticity of word reading. Visual and attentional processes in reading and dyslexia.

[bib59] Price C.J., Wise R.J.S., Frackowiak R.S.J. (1996). Demonstrating the implicit processing of visually presented words and pseudowords. Cerebral Cortex.

[bib60] Pulvermüller F. (1999). Words in the brain’s language. Behavioral and Brain Sciences.

[bib61] Pulvermüller F., Kujala T., Shtyrov Y., Simola J., Tiitinen H., Alku P., Alho K., Martinkauppi S., Ilmoniemi R.J., Näätänen R. (2001). Memory traces for words as revealed by the mismatch negativity. NeuroImage.

[bib62] Pulvermüller F., Shtyrov Y. (2006). Language outside the focus of attention: the mismatch negativity as a tool for studying higher cognitive processes. Progress in Neurobiology.

[bib63] Pulvermüller F., Shtyrov Y., Hasting A.S., Carlyon R.P. (2008). Syntax as a reflex: neurophysiological evidence for early automaticity of grammatical processing. Brain and Language.

[bib64] Pulvermüller F., Shtyrov Y., Hauk O. (2009). Understanding in an instant: neurophysiological evidence for mechanistic language circuits in the brain. Brain and Language.

[bib65] Pulvermüller F., Shtyrov Y., Ilmoniemi R. (2005). Brain signatures of meaning access in action word recognition. Journal of Cognitive Neuroscience.

[bib66] Ragazzoni A., Grippo A., Tozzi F., Zaccara G. (2000). Event-related potentials in patients with total locked-in state due to fulminant Guillain–Barre syndrome. International Journal of Psychophysiology.

[bib67] Rimon R., Fujita M., Takahata N. (1986). Mood alterations and sleep. Japanese Journal of Psychiatry and Neurology.

[bib68] Schröger E. (1996). On detection of auditory diviations: a pre-attentive activation model. Psychophysiology.

[bib69] Shewan C.M., Kertesz A. (1980). Reliability and validity characteristics of the western aphasia battery (WAB). Journal of Speech and Hearing Disorders.

[bib70] Shtyrov Y. (2010). Automaticity and attentional control in spoken language processing: neurophysiological evidence. Mental Lexicon.

[bib71] Shtyrov Y., Hauk O., Pulvermüller F. (2004). Distributed neuronal networks for encoding category-specific semantic information: the mismatch negativity to action words. European Journal of Neuroscience.

[bib72] Shtyrov Y., Kujala T., Pulvermüller F. (2010). Interactions between language and attention systems: early automatic lexical processing?. Journal of Cognitive Neuroscience.

[bib73] Shtyrov Y., Nikulin V.V., Pulvermüller F. (2010). Rapid cortical plasticity underlying novel word learning. Journal of Neuroscience.

[bib74] Shtyrov Y., Osswald K., Pulvermüller F. (2008). Memory traces for spoken words in the brain as revealed by the hemodynamic correlate of the mismatch negativity. Cerebral Cortex.

[bib75] Shtyrov Y., Pihko E., Pulvermüller F. (2005). Determinants of dominance: is language laterality explained by physical or linguistic features of speech?. NeuroImage.

[bib76] Shtyrov Y., Pulvermüller F. (2002). Neurophysiological evidence of memory traces for words in the human brain. NeuroReport.

[bib77] Shtyrov Y., Pulvermüller F. (2007). Early MEG activation dynamics in the left temporal and inferior frontal cortex reflect semantic context integration. Journal of Cognitive Neuroscience.

[bib78] Shtyrov Y., Pulvermüller F. (2007). Language in the mismatch negativity design: motivations, benefits and prospects. Journal of Psychophysiology.

[bib79] Sittiprapaporn W., Chindaduangratn C., Tervaniemi M., Khotchabhakdi N. (2003). Preattentive processing of lexical tone perception by the human brain as indexed by the mismatch negativity paradigm. Annals of the New York Academy of Sciences.

[bib80] Taulu S., Kajola M., Simola J. (2004). Suppression of interference and artifacts by the signal space separation method. Brain Topography.

[bib81] Taulu S., Simola J. (2006). Spatiotemporal signal space separation method for rejecting nearby interference in MEG measurements. Physics in Medicine and Biology.

[bib82] Tiitinen H., May P., Reinikainen K., Näätänen R. (1994). Attentive novelty detection in humans is governed by pre-attentive sensory memory. Nature.

[bib83] Worsley K.J., Taylor J.E., Tomaiuolo F., Lerch J. (2004). Unified univariate and multivariate random field theory. NeuroImage.

